# Supplementary material for: CAV2-expressing nerves induce metabolic switch toward mitochondrial oxidative phosphorylation to promote cancer stemness
Source: Nat Commun. 2025 Dec 2;17:203. doi: 10.1038/s41467-025-66914-2 (PMC12780207; doi:10.1038/s41467-025-66914-2)

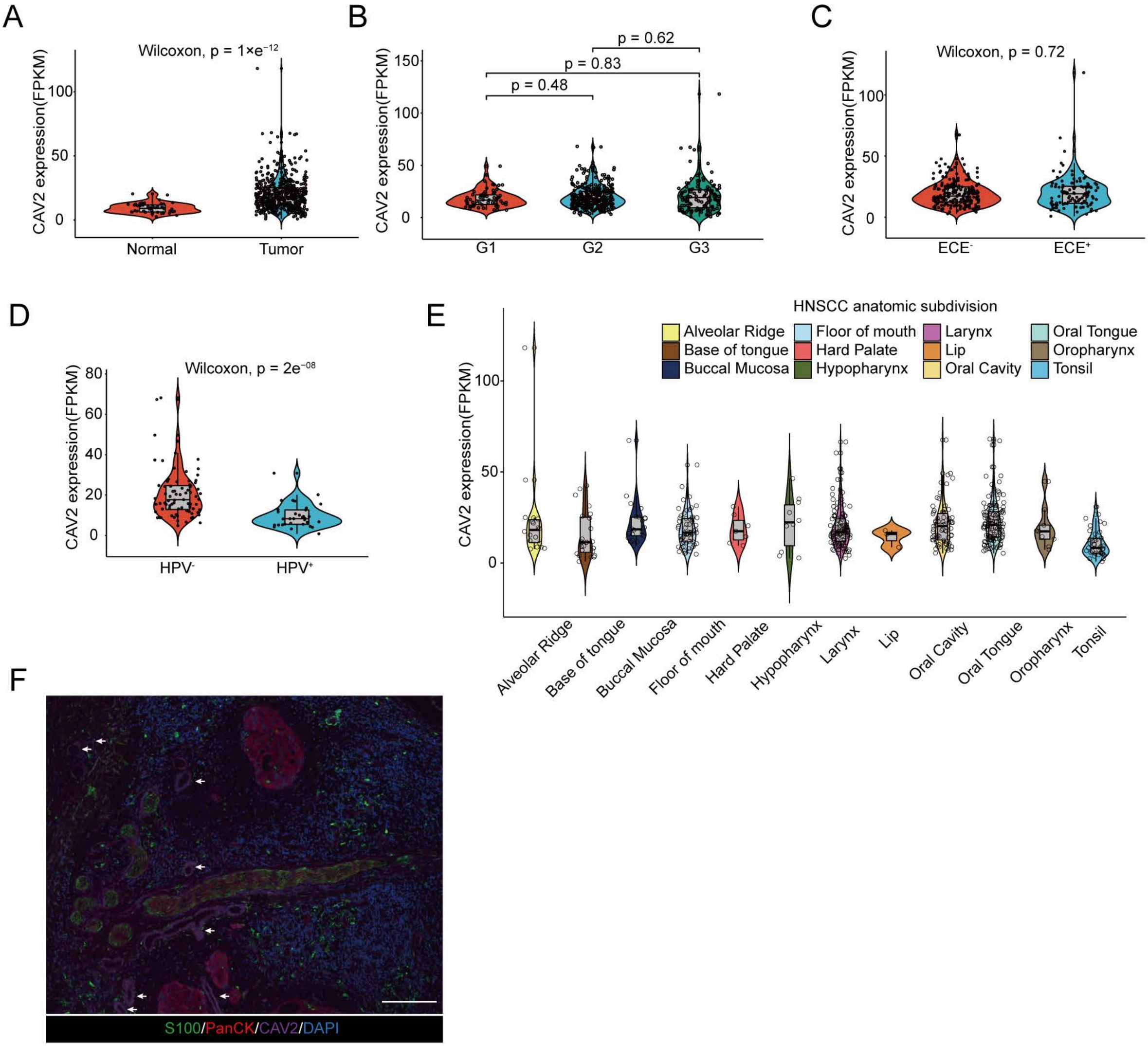
A
Wilcoxon, p = 1×e−12
CAV2 expression(FPKM)
Normal
Tumor
B
p = 0.62
p = 0.83
p = 0.48
G1
G2
G3
C
Wilcoxon, p = 0.72
ECE-
ECE+
D
Wilcoxon, p = 2e−08
HPV-
HPV+
E
HNSCC anatomic subdivision
Alveolar Ridge
Base of tongue
Buccal Mucosa
Floor of mouth
Hard Palate
Hypopharynx
Larynx
Lip
Oral Cavity
Oral Tongue
Oropharynx
Tonsil
F
S100/PanCK/CAV2/DAPI

Supplementary Figure 1 CAV2 Localization Patterns in HNSCC and Their Association with Clinical Outcomes.

(A) Elevated CAV2 expression in HNSCC (n = 500) relative to adjacent normal tissues (n = 44) in the TCGA cohort.

Utilizing sequencing data from the TCGA cohort's bulk tissue, correlations between CAV2 expression and clinicopathologic features, including (B) Histologic grade, (C) ECE, (D) HPV status, and (E) neoplasm's anatomical location are presented.

(F) Using Multiplex IHC with antibodies targeting CAV2, PanCK (epithelial marker), and S100 (neural marker), we discerned that in addition to nerve tissue, CAV2 was localized in vascular structures. Results were consistent across tissue sections from 15 patients. Scale: 200μm.

Violin plots show data distribution with the overlaid box plot indicating the median, first (Q1) and third (Q3) quartiles, and the whiskers extending to the 1.5x interquartile range (IQR). Two-sided statistical test.

HNSCC, head and neck squamous cell carcinoma; HPV, human papillomavirus; ECE, extracapsular extension

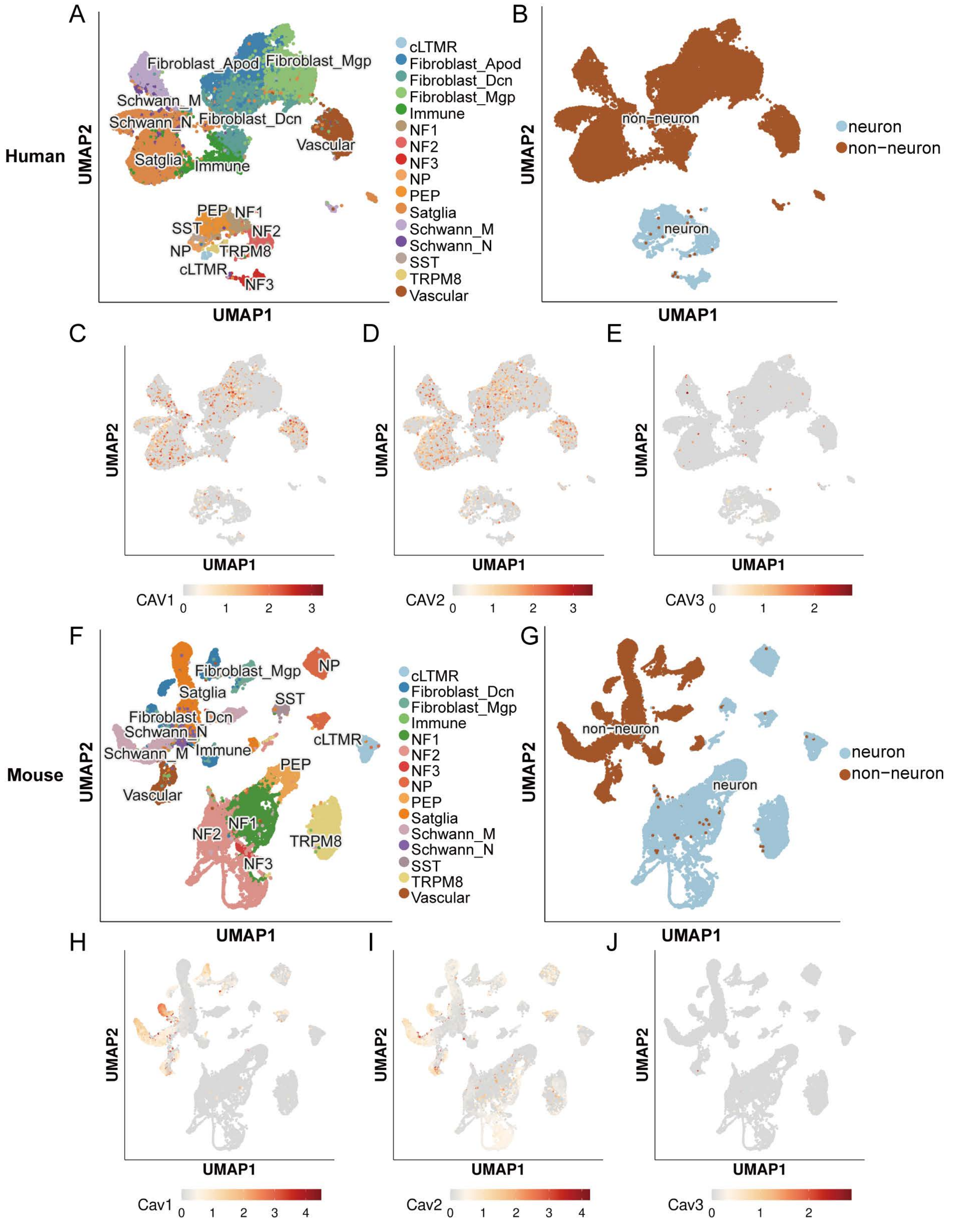

A
B
C
D
E
F
G
H
I
J
Human
Mouse
UMAP1
UMAP2
Fibroblast_Apod
Fibroblast_Mgp
Schwann_M
Schwann_N
Fibroblast_Dcn
Vascular
Satglia
Immune
PEP
NF1
SST
NF2
NP
TRPM8
cLTMR
NF3
cLTMR
Fibroblast_Apod
Fibroblast_Dcn
Fibroblast_Mgp
Immune
NF1
NF2
NF3
NP
PEP
Satglia
Schwann_M
Schwann_N
SST
TRPM8
Vascular
non−neuron
neuron
neuron
non−neuron
CAV1
CAV2
CAV3
Fibroblast_Dcn
Fibroblast_Mgp
Immune
Cav1
Cav2
Cav3

Supplementary Figure 2. Single-cell nuclear RNA sequencing analysis of human and mouse trigeminal ganglia reveals CAV1, CAV2, and CAV3 expression.

(A) UMAP plot depicting the clustering of single nuclei from human trigeminal ganglia (dataset GSE102994; https://painseq.shinyapps.io/tg-painseq/). Different cell types are color-coded and labeled, including various fibroblast subtypes (Apod, Dcn, Mgp), Schwann cells, neurons (NF1, NF2, NF3, PEP, NP, SST), satellite glia, immune cells, and vascular cells.

(B) UMAP plot of the same human trigeminal ganglia dataset, categorizing cells into neurons (blue) and non-neurons (brown).

(C–E) Feature plots illustrating the expression of CAV1 (C), CAV2 (D), and CAV3 (E) across the human trigeminal ganglia cell populations. The color intensity represents the relative level of gene expression.

(F) UMAP plot showing the clustering of single nuclei from mouse trigeminal ganglia.

(G) UMAP plot of mouse trigeminal ganglia data, categorizing cells into neurons (blue) and non-neurons (brown).

(H–J) Feature plots depicting the expression of Cav1 (H), Cav2 (I), and Cav3 (J) across the mouse trigeminal ganglia cell populations. The color intensity indicates the relative gene expression level.

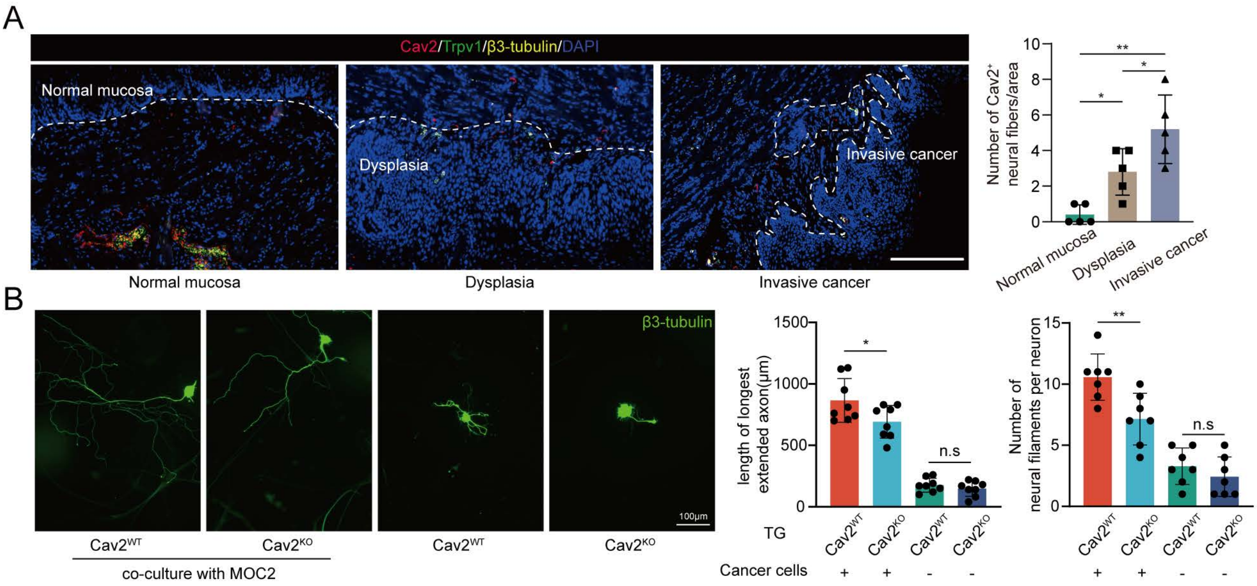
A
Cav2/Trpv1/β3-tubulin/DAPI
Normal mucosa
Dysplasia
Invasive cancer
Normal mucosa
Dysplasia
Invasive cancer
Number of Cav2⁺ neural fibers/area
10
8
6
4
2
0
**
*
*
Normal mucosa
Dysplasia
Invasive cancer
B
β3-tubulin
100μm
Cav2WT
Cav2KO
Cav2WT
Cav2KO
co-culture with MOC2
length of longest extended axon(μm)
1500
1000
500
0
*
n.s
TG
Cav2WT
Cav2KO
Cav2WT
Cav2KO
Cancer cells
+
+
-
-
Number of neural filaments per neuron
15
10
5
0
**
n.s
Cav2WT
Cav2KO
Cav2WT
Cav2KO
+
+
-
-

Supplementary Figure 3 Dynamics of Cav2+ nerves during 4-NQO–induced tumorigenesis and TG neuritogenesis post MOC2 co-culture.

(A) Left: Representative multicolor immunohistochemical images showing Cav2 (red), β3-tubulin (yellow), and Trpv1 (green) expression in tissue sections of 4-NQO induced murine HNSCC. Scale bar: 200 μm. Right: Quantitative analysis of densities of $Cav2^+$ nerves during various stages of 4-NQO-induced murine tumorigenesis. n=5 mice per group. line 1 vs line 2:*p=0.0238 (Two-sided Mann-Whitney U test), line 1 vs line 3:**p =0.0079 (Two-sided Mann-Whitney U test), line 2 vs line 3: *p=0.0497 (Two-sided Student's t-test). Data are mean ± s.d.

(B)Fluorescence images of TG neurons post-co-culture with MOC2 cells or controls. The subsequent quantification evaluates both neuritogenesis (n=7 biologically independent co-cultures per group) and the length of the most extended axons (n=8 per group), observed 72 hours post co-culture. *p=0.0449, **p=0.0078, Student's t-test (two-sided). Data are mean ± s.d.

TG, trigeminal ganglion; WT, wild-type; KO, knockout

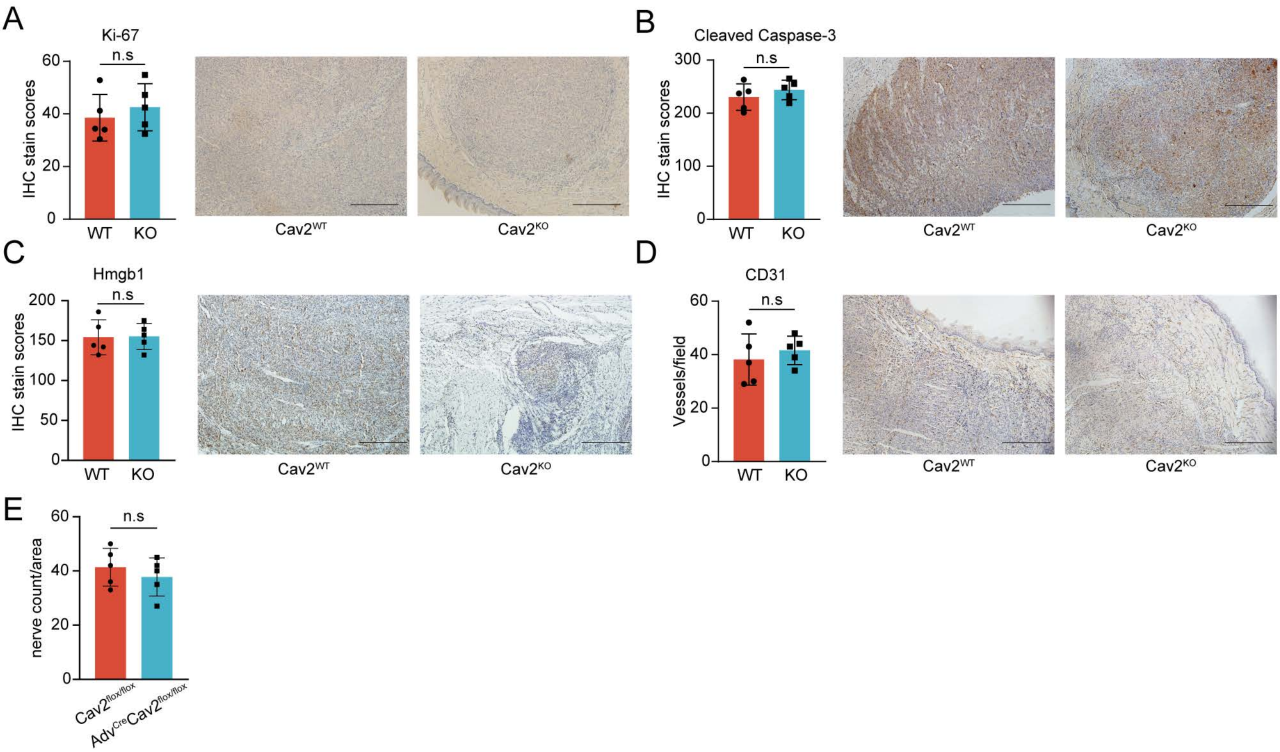

A
Ki-67
IHC stain scores
60
40
20
0
n.s
WT
KO
Cav2WT
Cav2KO
B
Cleaved Caspase-3
IHC stain scores
300
200
100
0
n.s
WT
KO
Cav2WT
Cav2KO
C
Hmgb1
IHC stain scores
200
150
100
50
0
n.s
WT
KO
Cav2WT
Cav2KO
D
CD31
Vessels/field
60
40
20
0
n.s
WT
KO
Cav2WT
Cav2KO
E
nerve count/area
60
40
20
0
n.s
Cav2flox/flox
AdvCreCav2flox/flox

Supplementary Figure 4

Quantitative analysis of IHC stain scores of (A)Ki-67, (B)Cleaved Caspase-3 and (C)Hmgb1. Scoring integrated tumor staining intensity and extent. Intensity: 0 (negative) to 3 (strongly positive). The final score resulted from multiplying intensity by the percentage of positive staining. Scale: 200μm. n=5 per group. n.s: (A)p=0.4975; (B) p=0.3622; (C) p=0.9368; Student's t-test(two-sided). Data are mean ± s.d.

(D) A quantitative assessment was performed to count $CD31^+$ angiogenic vessels in each visual field. Scale: 200μm. n=5 per group. n.s: p=0.5073, Student's t-test(two-sided). Data are mean ± s.d.

(E) Quantification of nerve numbers in mice from different groups. n.s: p=0.4408, Student's t-test(two-sided). Data are mean ± s.d.

WT: Wild-Type; KO: Knockout; IHC: immunohistochemistry

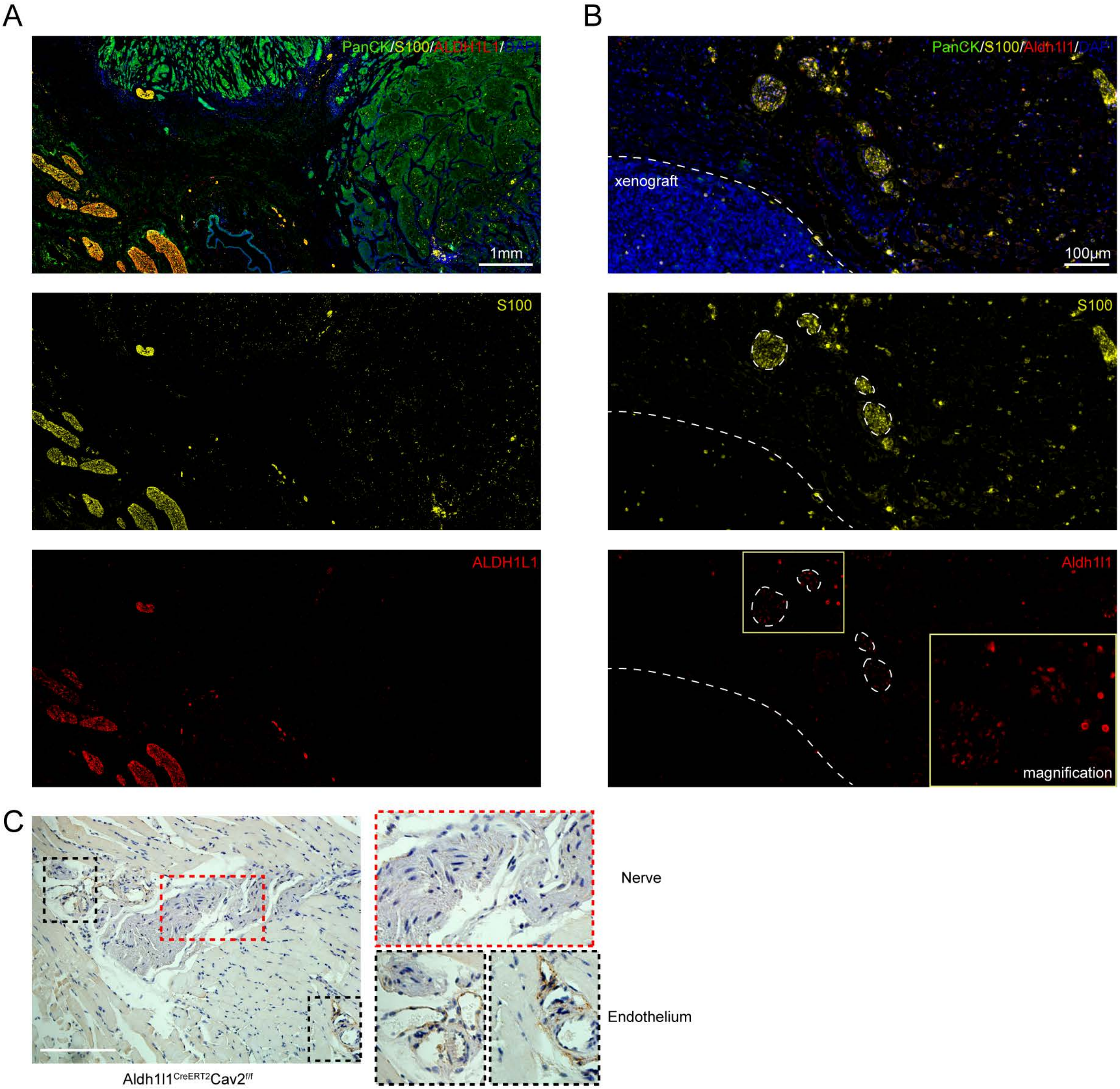
A
PanCK/S100/ALDH1L1/DAPI
1mm
S100
ALDH1L1
B
PanCK/S100/Aldh1l1/DAPI
xenograft
100μm
S100
Aldh1l1
magnification
C
Aldh1l1CreERT2Cav2f/f
Nerve
Endothelium

Supplementary Figure 5

(A) Multicolor IHC of human HNSCC tissue sections shows ALDH1L1 expression (red) is specific to Schwann cells, as indicated by co-staining with S100 (yellow) and exclusion from PanCK-positive epithelial cells (green). Results were consistent across tissue sections from 5 patients. (B) Similar multicolor IHC in murine orthotopic tongue tumor sections confirms Aldh1l1 expression (red) is restricted to Schwann cells ($S100^{+}$, yellow; $PanCK^{-}$, green). Results were consistent across tissue sections from 5 mice. (C) IHC analysis of $Aldh1l1^{CreERT2}Cav2^{flox/flox}$ mouse tongue tissue reveals a lack of Cav2 expression in neural tissue, while endothelial cells retain normal Cav2 expression. Results were consistent across tissue sections from 4 mice. Scale: 100µm.

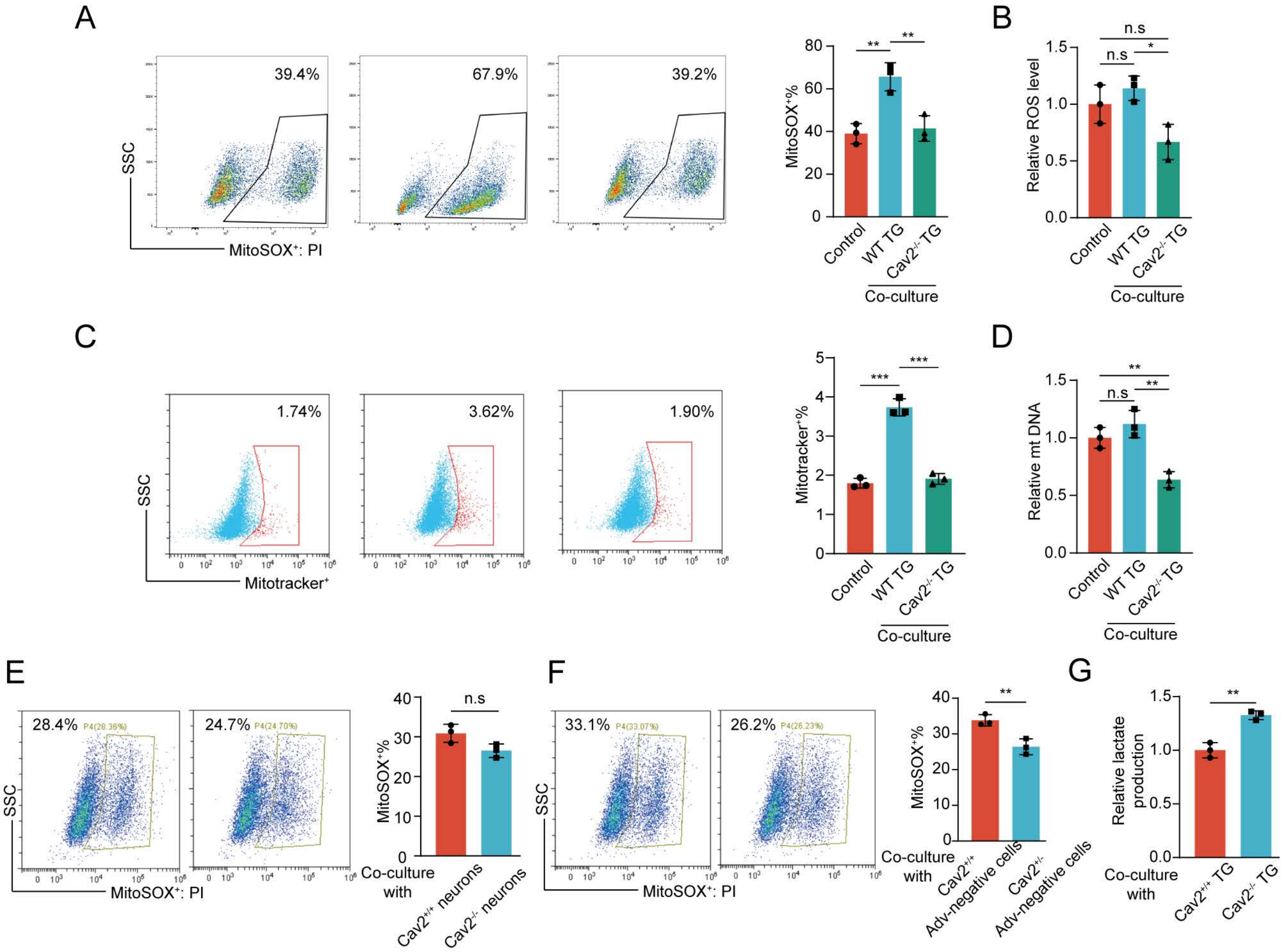
A
39.4%
67.9%
39.2%
SSC
MitoSOX⁺: PI
MitoSOX⁺%
**
**
Control
WT TG
Cav2-/- TG
Co-culture
B
n.s
n.s
*
Relative ROS level
Control
WT TG
Cav2-/- TG
Co-culture
C
1.74%
3.62%
1.90%
SSC
Mitotracker⁺
Mitotracker⁺%
***
***
Control
WT TG
Cav2-/- TG
Co-culture
D
**
n.s
**
Relative mt DNA
Control
WT TG
Cav2-/- TG
Co-culture
E
28.4%
24.7%
SSC
MitoSOX⁺: PI
MitoSOX⁺%
n.s
Co-culture with
Cav2+/+ neurons
Cav2-/- neurons
F
33.1%
26.2%
SSC
MitoSOX⁺: PI
MitoSOX⁺%
**
Co-culture with
Cav2+/+ Adv-negative cells
Cav2-/- Adv-negative cells
G
**
Relative lactate production
Co-culture with
Cav2+/+ TG
Cav2-/- TG

Supplementary Figure 6

(A) MitoSOX Red staining and flow cytometry analysis of MOC2 cells after 72-hour co-culture conditions. n=3 biologically independent experiments per group. line 1 vs line2: **p=0.0047; line 2 vs line 3: **p=0.0093, Student's t-test (two-sided). Data are mean ± s.d.

(B) ROS-Glo determination of ROS levels of MOC2 cells. n=3 biologically independent experiments per group. *p=0.0123, Student's t-test (two-sided). Data are mean ± s.d.

(C) MOC2 cells from different co-culture conditions stained with MitoTracker Red CMXRos and analyzed via flow cytometry. n=3 biologically independent experiments per group. line 1 vs line 2: ***p=0.0002; line 2 vs line 3: ***p=0.0003. Student's t-test (two-sided). Data are mean ± s.d.

(D) Mitochondrial DNA levels in MOC2 cells ascertained via real-time qPCR. n=3 biologically independent experiments per group. line1 vs line 3: **p=0.0053, line2 vs line 3: **p=0.0037. Student's t-test (two-sided). Data are mean ± s.d.

(E) TG from $Cav2^{+/+}$ and $Cav2^{-/-}$ mice were enzymatically dissociated, and glial cell growth was inhibited by cytosine arabinoside treatment. Co-culture with SCC15 cells was performed, followed by MitoSOX Red staining and flow cytometry analysis to evaluate SCC15 cells. n=3 biologically independent experiments per group. n.s, p=0.0568, Student's t-test (two-sided). Data are mean ± s.d.

(F) Trigeminal ganglia from $mTmG^{+/-}$; Adv-Cre; $Cav2^{+/-}$ and $mTmG^{+/-}$; Adv-Cre; $Cav2^{+/+}$ mice were sorted for Adv-negative cells via flow cytometry and then co-

cultured with SCC15 cells. MitoSOX Red staining and flow cytometry analysis were performed to evaluate SCC15 cells. n=3 biologically independent experiments per group. **p=0.0091, Student's t-test (two-sided). Data are mean ± s.d.

(G) Quantification of lactate secretion levels in SCC15 cells co-cultured with $Cav2^{+/+}$ or $Cav2^{-/-}$ trigeminal ganglia. n=3 biologically independent experiments per group. **p=0.0023, Student's t-test (two-sided). Data are mean ± s.d.

TG, trigeminal ganglion; ROS, reactive oxygen species; WT, wild type

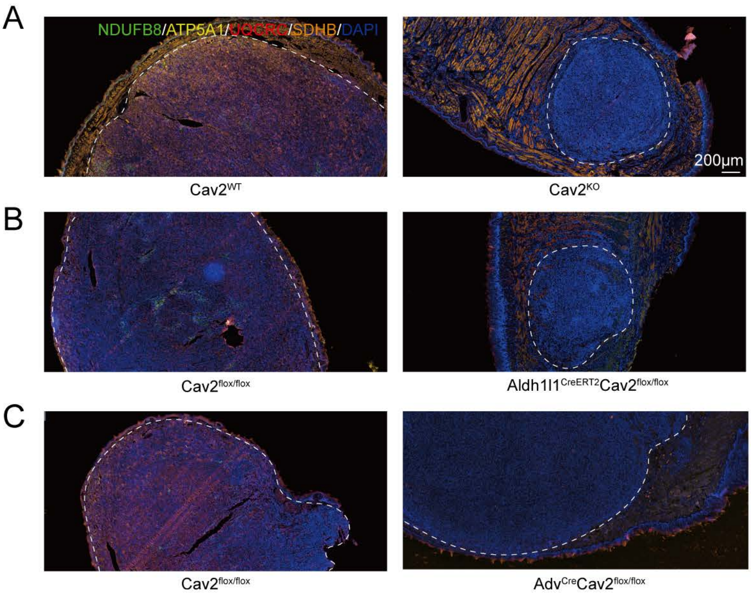
A
NDUFB8/ATP5A1/UQCRC/SDHB/DAPI
200μm
Cav2WT
Cav2KO
B
Cav2flox/flox
Aldh1l1CreERT2Cav2flox/flox
C
Cav2flox/flox
AdvCreCav2flox/flox

Supplementary Figure 7 Mitochondrial respiratory complex markers in various Cav2-deficient murine lines.

As shown, NDUFB8, ATP5A1, UQCRC, and SDHB were analyzed by multicolor immunohistochemistry in (A) $Cav2^{+/+}$ and $Cav2^{-/-}$ mice, (B) $Aldh1l1^{CreERT2}Cav2^{f/f}$ and $Cav2^{f/f}$ mice, and (C) $Adv^{Cre}Cav2^{f/f}$ and $Cav2^{f/f}$ mice. Results were consistent across tissue sections from 5 mice in each group.

WT, wild-type; KO, knockout

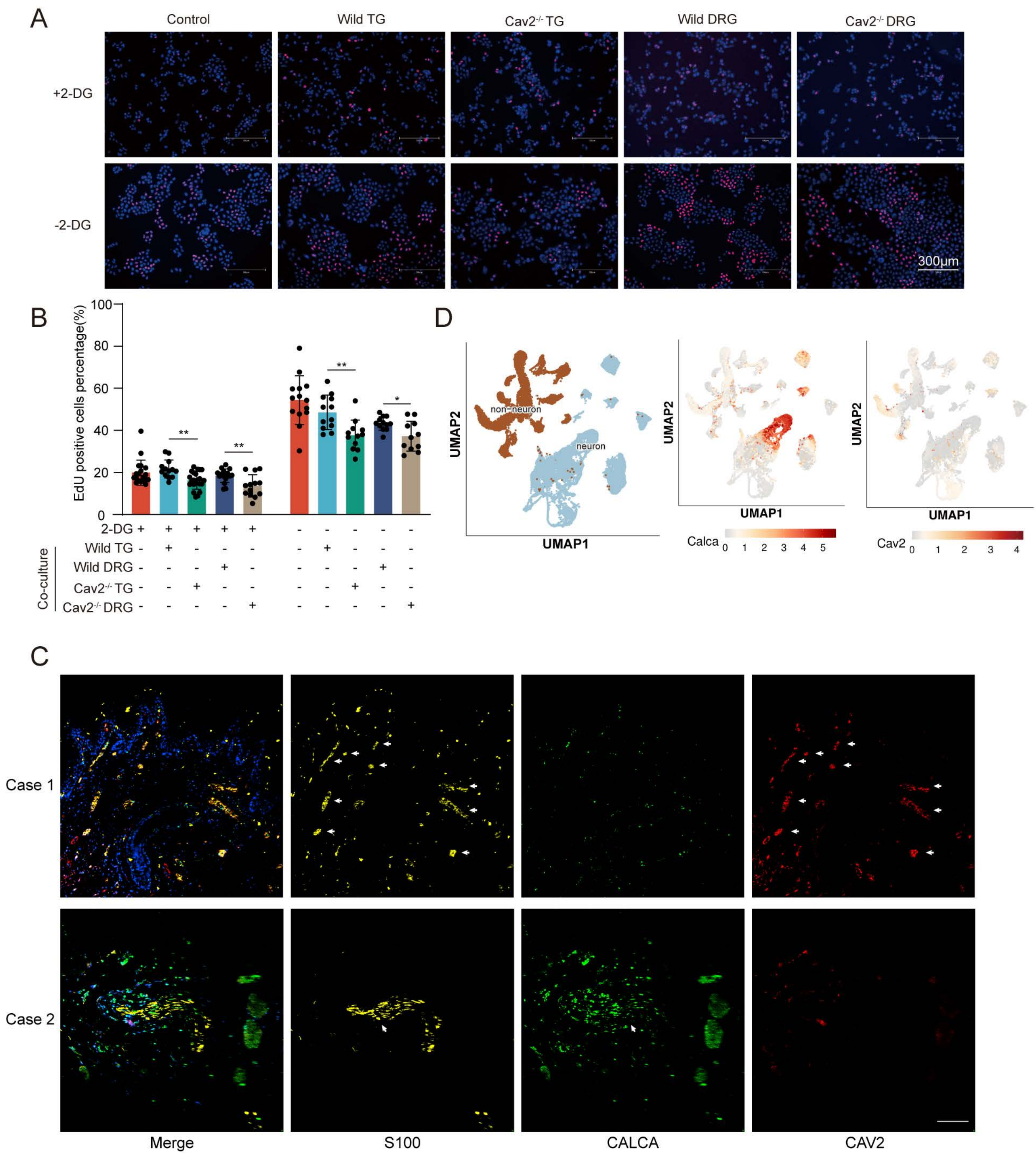

A
Control
Wild TG
Cav2-/- TG
Wild DRG
Cav2-/- DRG
+2-DG
-2-DG
300μm
B
EdU positive cells percentage(%)
100
80
60
40
20
0
**
**
**
*
2-DG
Wild TG
Wild DRG
Cav2-/- TG
Cav2-/- DRG
Co-culture
D
non−neuron
neuron
UMAP2
UMAP1
Calca
0 1 2 3 4 5
Cav2
0 1 2 3 4
C
Case 1
Case 2
Merge
S100
CALCA
CAV2

Supplementary Figure 8 Impact of neural Cav2 on HNSCC cell proliferation and the distinct expression profiles of Cav2 and Calca.

(A) Proliferation of SCC15 cells co-cultured with TG or DRG assessed with an EdU assay, with or without 2-DG treatment.

(B) EdU-positive cells were counted and represented as a fraction of every 100 cells. with n=19 biologically independent cell cultures for group one, n=13 for group two, n=21 for group three, n=16 for group four, n=14 for group five, n=12 for group six, n=12 for group seven, and n=12 for group eight. line 2 vs line 3: **p=0.0011; line 4 vs line 5: **p=0.0049; line 7 vs line 8: **p=0.0023; line 9 vs line 10: *p=0.0130, Student's t-test (two-sided). Data are mean ± s.d.

(C) The upper row shows cases where neural tissue expresses Cav2 but lacks significant Calca expression, while the lower row shows cases with Calca expression but no significant Cav2 expression. Each expression pattern was observed in tissue sections from 5 patients. Scale: 100μm.

(D) Single-nucleus RNA sequencing of the trigeminal ganglion (dataset GSE102994) reveals discordant expression patterns of Cav2 and Calca.

TG, trigeminal ganglion; DRG, dorsal root ganglion

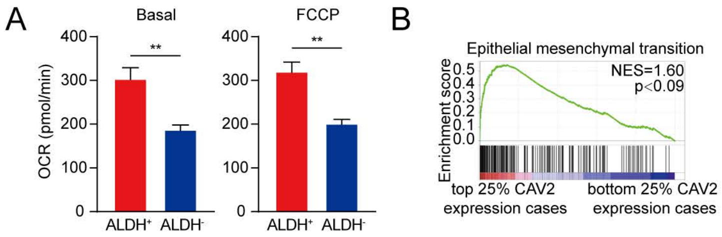
A
Basal
FCCP
OCR (pmol/min)
400
300
200
100
0
**
ALDH+ ALDH-
B
Epithelial mesenchymal transition
Enrichment score
NES=1.60
p<0.09
0.5
0.4
0.3
0.2
0.1
0.0
top 25% CAV2 expression cases
bottom 25% CAV2 expression cases

Supplementary Figure 9

(A) OCRs were evaluated under basal conditions and upon FCCP treatment. n=3 biologically independent experiments per group. Basal: **p=0.0029; FCCP: **p=0.0016. Student's t-test(two-sided) . Data are mean ± s.d.

(B) Within the TCGA HNSCC project, patients were categorized into quartiles based on CAV2 expression levels, focusing on the highest and lowest 25%. Analysis through GSEA demonstrated that the 'HALLMARK_Epithelial_Mesenchymal_Transition' gene set was distinctly enriched in the cohort with elevated CAV2 expression.

OCR, oxygen consumption rate; HNSCC, head and neck squamous cell carcinoma; FCCP, carbonyl cyanide p-trifluoromethoxyphenylhydrazone

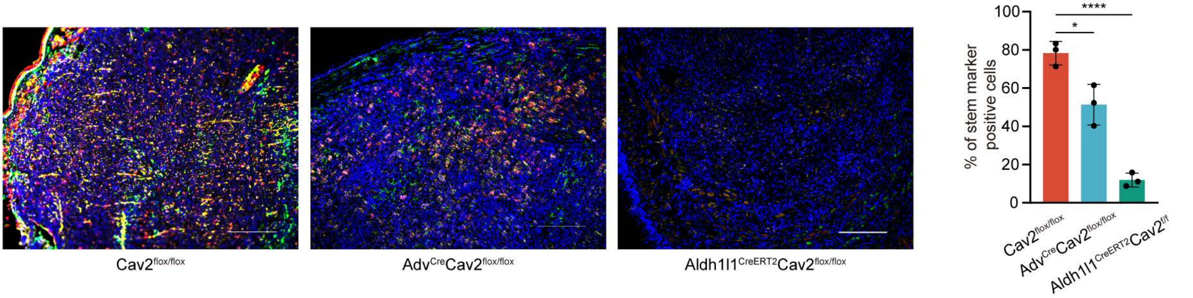
Cav2flox/flox
AdvCreCav2flox/flox
Aldh1l1CreERT2Cav2flox/flox
% of stem marker positive cells
100
80
60
40
20
0
****
*
Cav2flox/flox
AdvCreCav2flox/flox
Aldh1l1CreERT2Cav2f/f

Supplementary Figure 10

Representative multicolor immunohistochemical staining for Aldh, CD44, and Bmi1 in the tongues of MOC2-engrafted mice, comparing $Cav2^{f/f}$ with $Adv^{Cre}Cav2^{f/f}$ mice and $Aldh1l1^{CreERT2}Cav2^{f/f}$ mice. Scale: 200µm. n=3 mice per group. *p=0.0192, ****$p < 0.0001$, Student's t-test (two-sided). Data are mean ± s.d.

Supplementary dataset. Full GSEA output for HNSCC cells co-cultured with wild-type versus Cav2$^{-/-}$ trigeminal ganglia.

uncropped western blot-fig. 2A

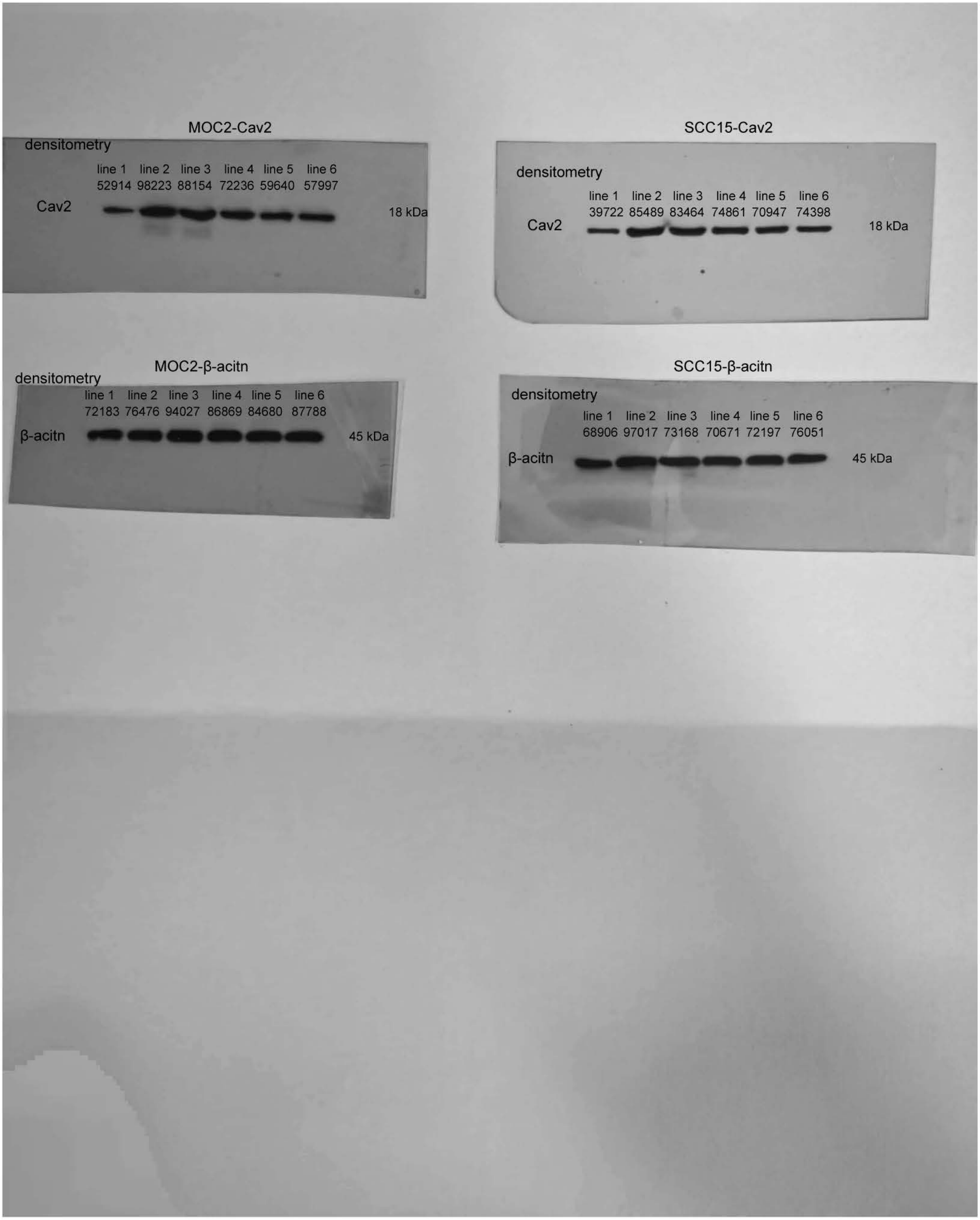

uncropped western blot-fig. 3E-F

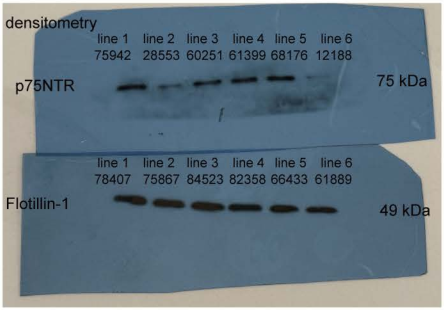

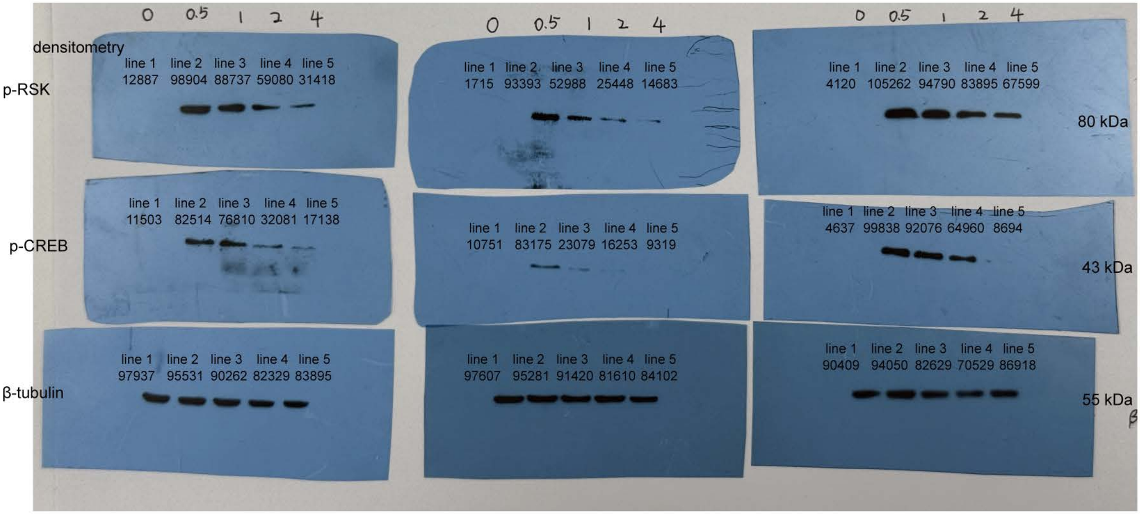

Supplement: Supplementary file 1 — Supplementary Information [file 41467_2025_66914_MOESM1_ESM.pdf]
