# Supplementary material for: CAV2-expressing nerves induce metabolic switch toward mitochondrial oxidative phosphorylation to promote cancer stemness
Source: Nat Commun. 2025 Dec 2;17:203. doi: 10.1038/s41467-025-66914-2 (PMC12780207; doi:10.1038/s41467-025-66914-2)
Supplement: Supplementary file 2 — Description of Additional Supplementary Files [file 41467_2025_66914_MOESM2_ESM.pdf]

Description of Additional Supplementary Files

Supplementary Data 1. Full GSEA output for HNSCC cells co-cultured with wild-type versus $Cav2^{-/-}$ trigeminal ganglia.
