# Supplementary material for: CAV2-expressing nerves induce metabolic switch toward mitochondrial oxidative phosphorylation to promote cancer stemness
Source: Nat Commun. 2025 Dec 2;17:203. doi: 10.1038/s41467-025-66914-2 (PMC12780207; doi:10.1038/s41467-025-66914-2)
Supplement: Supplementary file 4 — Reporting Summary [file 41467_2025_66914_MOESM4_ESM.pdf]

nature portfolio

Corresponding author(s): Hua Guo, Ze Zhang, Ruoyan Liu, Jingtao Luo

Last updated by author(s): Nov 11, 2025

Reporting Summary

Nature Portfolio wishes to improve the reproducibility of the work that we publish. This form provides structure for consistency and transparency in reporting. For further information on Nature Portfolio policies, see our Editorial Policies and the Editorial Policy Checklist.

Statistics

For all statistical analyses, confirm that the following items are present in the figure legend, table legend, main text, or Methods section.

n/a	Confirmed	
☐	☒	The exact sample size (*n*) for each experimental group/condition, given as a discrete number and unit of measurement
☐	☒	A statement on whether measurements were taken from distinct samples or whether the same sample was measured repeatedly
☐	☒	The statistical test(s) used AND whether they are one- or two-sided<br>*Only common tests should be described solely by name; describe more complex techniques in the Methods section.*
☐	☒	A description of all covariates tested
☐	☒	A description of any assumptions or corrections, such as tests of normality and adjustment for multiple comparisons
☐	☒	A full description of the statistical parameters including central tendency (e.g. means) or other basic estimates (e.g. regression coefficient) AND variation (e.g. standard deviation) or associated estimates of uncertainty (e.g. confidence intervals)
☐	☒	For null hypothesis testing, the test statistic (e.g. *F*, *t*, *r*) with confidence intervals, effect sizes, degrees of freedom and *P* value noted<br>*Give P values as exact values whenever suitable.*
☒	☐	For Bayesian analysis, information on the choice of priors and Markov chain Monte Carlo settings
☒	☐	For hierarchical and complex designs, identification of the appropriate level for tests and full reporting of outcomes
☒	☐	Estimates of effect sizes (e.g. Cohen's *d*, Pearson's *r*), indicating how they were calculated

Our web collection on statistics for biologists contains articles on many of the points above.

Software and code

Policy information about availability of computer code

Data collection	Data was collected using Microsoft Excel 2016 64-bit (Microsoft), ZEN 2.3(ZEISS), DPManager (Olympus), EVOS M5000 (Invitrogen), Gen5 (BioTek Instruments), EMIP(Hitachi), Wave Desktop Software (Agilent Technologies), Wave Desktop Software (Agilent Technologies), BZ-X Analyzer (KEYENCE), GV6000 PLUS (BLT) and NANODROP 2000/2000c (Thermo Fisher Scientific).
Data analysis	Data was analyzed using GraphPad Prism Version 9.5.1, the R language environment for statistical computing version 4.0.3, imageJ 1.52 (NIH, USA), GSEA 4.1.0(BROAD INSTITUTE), FACS Diva Software Version 8.0.1 (BD Biosciences), CytoFLEX Software (Beckman Coulter), ZEN 2.3 (ZEISS), DPManager(Olympus), EVOS M5000 (Invitrogen), Gen5 (BioTek Instruments), EMIP(Hitachi), Wave Desktop Software (Agilent Technologies) and BZ-X Analyzer (KEYENCE). Western blots were cropped in Adobe Photoshop CS6. The figures in the paper were composed using Adobe Illustrator CC 2015.

For manuscripts utilizing custom algorithms or software that are central to the research but not yet described in published literature, software must be made available to editors and reviewers. We strongly encourage code deposition in a community repository (e.g. GitHub). See the Nature Portfolio guidelines for submitting code & software for further information.

Data

Policy information about availability of data

All manuscripts must include a data availability statement. This statement should provide the following information, where applicable:

- Accession codes, unique identifiers, or web links for publicly available datasets
- A description of any restrictions on data availability
- For clinical datasets or third party data, please ensure that the statement adheres to our policy

Source data for the figures and extended data figures are provided. The datasets generated during the current study are available. The sequencing data have been deposited at the Gene Expression Omnibus under accession number GSE288675. The mass spectrometry data are publicly available in the PRIDE database under accession number PXD060212.

Research involving human participants, their data, or biological material

Policy information about studies with human participants or human data. See also policy information about sex, gender (identity/presentation), and sexual orientation and race, ethnicity and racism.

Reporting on sex and gender	In the 101 patients included in this study, 73 were male and 28 were female. This is attributed to the higher incidence rate of head and neck squamous carcinoma in the male population.
Reporting on race, ethnicity, or other socially relevant groupings	The cases included in this study are all of Asian ethnicity, which is determined by the geographical location of the researchers' institution.
Population characteristics	Investigating the role of neural CAV2 expression in determining patient outcomes, our study encompassed individuals diagnosed with head and neck squamous cell carcinoma, averaging 58 years in age. All participants provided informed consent prior to their inclusion in the study. No compensation was offered to participants, as the study did not result in any adverse effects.
Recruitment	This study utilized tumor specimens from patients treated within the Department of Head and Neck Surgical Oncology at Tianjin Medical University Cancer Institute & Hospital, Tianjin, China, from 2010 to 2015.
Ethics oversight	The study was conducted with approval from the ethics committee of Tianjin Cancer Hospital.

Note that full information on the approval of the study protocol must also be provided in the manuscript.

Field-specific reporting

Please select the one below that is the best fit for your research. If you are not sure, read the appropriate sections before making your selection.

☒ Life sciences ☐ Behavioural & social sciences ☐ Ecological, evolutionary & environmental sciences

For a reference copy of the document with all sections, see nature.com/documents/nr-reporting-summary-flat.pdf

Life sciences study design

All studies must disclose on these points even when the disclosure is negative.

Sample size	Samples sizes from other experiments were estimated from similar experiments in former publications of the group. Sample sizes were arrived based on having at least three independent biological replicates.<br>Sample sizes for clinical data:<br>No statistical methods were used to predetermine sample sizes and samples were selected based upon the availability of data as outlined below.<br>-TCGA cohorts of head and neck squamous cell carcinoma were assessed.<br>-The expression and localization of CAV2, TH, and TRPV1 in glossectomy tissues from treatment-naïve patients with HNSCC treated at the Tianjin Cancer Hospital, Tianjin, China were analyzed (n = 12).<br>-The expression levels of neural CAV2 within tumor areas in patients with HNSCC was evaluated and compared with their survival (n = 101).<br><br>In vitro studies:<br>sample sizes were determined based on the results of pilot studies, and previous similar studies that have given statistically significant results.<br>In vivo studies:<br>Animal experiments were conducted using between 5 and 8 mice per group, based on the results of pilot studies and previous studies.
Data exclusions	No data excluded.
Replication	All experimental replications were successfully conducted. These experiments were executed a minimum of twice, and with an adequate number of cells or animals per group to ensure statistical significance. The specific number of replicates for each experiment is detailed in the respective figure legends
Randomization	In this study, most of the animal experiments were not randomly grouped, as the grouping was predominantly determined by the genotype of

the mice, such as wild-type versus CAV-KO.

Blinding	The researchers were blinded during the measurement of the tumor size and body weight of mice.<br>The researchers were blinded during IHC outcome assessment.

Reporting for specific materials, systems and methods

We require information from authors about some types of materials, experimental systems and methods used in many studies. Here, indicate whether each material, system or method listed is relevant to your study. If you are not sure if a list item applies to your research, read the appropriate section before selecting a response.

Materials & experimental systems

n/a	Involved in the study
☐	☒ Antibodies
☐	☒ Eukaryotic cell lines
☒	☐ Palaeontology and archaeology
☐	☒ Animals and other organisms
☐	☒ Clinical data
☒	☐ Dual use research of concern
☒	☐ Plants

Methods

n/a	Involved in the study
☒	☐ ChIP-seq
☐	☒ Flow cytometry
☒	☐ MRI-based neuroimaging

Antibodies

Antibodies used

IHC assay:
anti-CAV2 (NBP1-31116, Novus, Lot:39778)(1:200)
anti- S100 (15146-1-AP, Proteintech, Lot:00120806)(1:2000)
anti-panCK (#ab9377, Abcam, Lot:GR3377716-3)(1:100)
anti-Ki67 (#12202, Cell Signaling Technology)(1:200)
anti-TH (25859-1-AP, Proteintech, Lot:00096177)(1:1000)
anti-TRPV1 (ACC-030, Alomone labs, Lot:ACC030AN3602)(1:200)
anti-β3-tubulin (#ab52623, Abcam, Lot:GR3416293-3)(1:100)
anti-ALDH1A1 (15910-1-AP, Proteintech, Lot:00105494)(1:100)
and anti-CD44 (15675-1-AP, Proteintech, Lot:00118645)(1:1000)
anti-pS6 (#4858, Cell Signaling Technology, Lot:21)(1:400)
anti-CD31(#ab281583, Abcam)(1:4000)
anti-Cleaved Caspase-3(#9664, Cell Signaling Technology, Lot:22)(1:2000)
anti-BMI1 (10832-1-AP, Proteintech, Lot:00050535)(1:300)
anti-ALDH1L1 (#ab235197, Abcam)(1:200)
anti-NDUFB8 (14794-1-AP, Proteintech,Lot:00120885)(1:100)
anti-ATP5A1 (14676-1-AP, Proteintech,Lot:00126035)(1:500)
anti-SDHB (10620-1-AP, Proteintech,Lot:00121226)(1:200)
anti-UQCRC1 (21705-1-AP, Proteintech,Lot:00040540)(1:200)

Immunofluorescence:
anti-CAV2 (NBP1-31116, Novus, Lot:39778) (1:200)
anti-β3-tubulin (ab52623, Abcam, Lot:GR3416293-3)(1:500)
FITC-conjugated secondary antibody (A21206, Invitrogen, Lot:2376850)(2 μg/mL)

Western blot:
anti-CAV2 (NBP1-31116, Novus, Lot:39778) (1:500)
anti-β-actin(#4967, Cell Signaling Technology, Lot:13)(1:1000)
anti-p-RSK(sc-377501, Santa Cruz Biotechnology)(1:100)
anti-p-CREB(#9198, Cell Signaling Technology)(1:500)
anti-β-tubulin(#2146, Cell Signaling Technology)(1:1000)
anti-p75NTR(#8238, Cell Signaling Technology)(1:1000)
anti-Flotillin-1(#3253, Cell Signaling Technology) (1:1000)

Validation

All primary antibodies used in this study were highly validated and sourced from reputable commercial suppliers, including Abcam, Novus, Alomone Labs, Proteintech, and Cell Signaling Technology. These antibodies were employed at concentrations recommended by the manufacturers.

Eukaryotic cell lines

Policy information about cell lines and Sex and Gender in Research

Cell line source(s)	SCC15 cells were procured from the ATCC(CRL-1623). PC-12 cells were procured from the National Collection of Authenticated Cell Cultures(TCR 9). MOC1 and MOC2 cells were procured from the BLUEFBIO(BFN6021632 and BFN6021637).
Authentication	All cell lines are subject to STR authentication by the selling institution prior to sale.
Mycoplasma contamination	All cell lines underwent routine testing for mycoplasma contamination, which included immediate testing upon receipt. Consistently, all tests yielded negative results for mycoplasma contamination, confirming the purity of the cell cultures.
Commonly misidentified lines (See ICLAC register)	No commonly misidentified cell lines were used in this study.

Animals and other research organisms

Policy information about studies involving animals; ARRIVE guidelines recommended for reporting animal research, and Sex and Gender in Research

Laboratory animals	Cav2+/- mice were procured from Cyagen Biosciences (S-KO-01350, Cyagen Biosciences).<br>Cav2flox/+ mice were procured from Cyagen Biosciences (S-CKO-01562, Cyagen Biosciences).<br>Aldh1l1-CreERT2 mice were procured from Cyagen Biosciences (C001288, Cyagen Biosciences).<br>Adv-Cre mice were procured from SHANG HAI MODEL ORGANISMS (NM-KI-215036, SHANG HAI MODEL ORGANISMS).<br>mTmG mice were procured from Jackson lab (007676, Jackson lab). The genetic background of all mice was C57BL/6. All mice used in the animal experiments of this study were aged 6–10 weeks, including those administered with 4-NQO at the initiation of tumor induction(This excludes mice from which trigeminal ganglia (TG) and dorsal root ganglia (DRG) were harvested for in vitro experiments). In this study, the gender of the mice was determined by the sex of the offspring produced through mating and breeding, rather than being pre-selected. The sex of each mouse is indicated in Figure 4, and no significant phenotypic differences were observed between males and females.
Wild animals	No wild animals were used.
Reporting on sex	In this study, the gender of the mice used typically depends on the gender of the offspring obtained after mating and breeding, rather than a pre-designed gender.
Field-collected samples	No field-collected samples were used.
Ethics oversight	Study protocols were approved by the Animal Ethical and Welfare Committee of Tianjin Medical University Cancer Institute and Hospital.

Note that full information on the approval of the study protocol must also be provided in the manuscript.

Clinical data

Policy information about clinical studies

All manuscripts should comply with the ICMJE guidelines for publication of clinical research and a completed CONSORT checklist must be included with all submissions.

Clinical trial registration	NA
Study protocol	NA
Data collection	This study utilized tumor specimens from patients treated at the Department of Head and Neck Surgical Oncology, Tianjin Medical University Cancer Institute & Hospital (TJCH cohort), Tianjin, China, between 2010 and 2015. A total of 101 patients (73 males and 28 females) were included. Patients were selected based on the availability of formalin-fixed paraffin-embedded (FFPE) tumor specimens and corresponding clinical and survival data. The inclusion criteria required complete clinical information and follow-up data. Potential biases, such as self-selection bias, were minimized as eligible patients meeting the criteria during the study period were included. However, as this is a single-center, retrospective study, there may be inherent selection bias based on the institution's patient population and the availability of follow-up data. This study was conducted in accordance with ethical guidelines and approved by the Tianjin Medical University Cancer Institute and Hospital Institutional Review Board (IRB).
Outcomes	NA

Plants

Seed stocks

Report on the source of all seed stocks or other plant material used. If applicable, state the seed stock centre and catalogue number. If plant specimens were collected from the field, describe the collection location, date and sampling procedures.

Novel plant genotypes

Describe the methods by which all novel plant genotypes were produced. This includes those generated by transgenic approaches, gene editing, chemical/radiation-based mutagenesis and hybridization. For transgenic lines, describe the transformation method, the number of independent lines analyzed and the generation upon which experiments were performed. For gene-edited lines, describe the editor used, the endogenous sequence targeted for editing, the targeting guide RNA sequence (if applicable) and how the editor was applied.

Authentication

Describe any authentication procedures for each seed stock used or novel genotype generated. Describe any experiments used to assess the effect of a mutation and, where applicable, how potential secondary effects (e.g. second site T-DNA insertions, mosiacism, off-target gene editing) were examined.

Flow Cytometry

Plots

Confirm that:

- [x] The axis labels state the marker and fluorochrome used (e.g. CD4-FITC).
- [x] The axis scales are clearly visible. Include numbers along axes only for bottom left plot of group (a 'group' is an analysis of identical markers).
- [ ] All plots are contour plots with outliers or pseudocolor plots.
- [x] A numerical value for number of cells or percentage (with statistics) is provided.

Methodology

Sample preparation

The ALDEFLUOR kit (# 01700, Stem Cell Technologies) was employed, following the manufacturer's guidelines, to gauge ALDH1 activity within cancer cells and sort cell groups exhibiting elevated ALDH1 activity. For mitochondrial assessment, cells were stained with 10 nM MitoTracker Red CMXRos (M7512, Invitrogen) under conditions of room temperature for 20 minutes or 5 µM Mitosox (M36008, Invitrogen) under conditions of 37°C and 5% CO2 for 30 minutes. For the apoptosis assay, HNSCC cells, whether co-cultured with wild-type TG or Cav2-/- TG, underwent treatment with the specified concentration of cisplatin, followed by dissociation using 0.25% trypsin and subsequent collection via centrifugation. The cells were then subjected to a 30-minute staining procedure at 4°C utilizing the apoptosis kit from BioLegend, in compliance with the manufacturer's prescribed protocol.

Instrument

The BD LSRFortessa X-20 Cell Analyzer (BD Biosciences) and the CytoFLEX LX Cell Analyzer (Beckman Coulter) were utilized for all assay analyses in this study.

Software

BD FACS Diva Software Version 8.0.1 and CytoFLEX Software were used to collect the data.
FlowJo Version 1.0.1 was used to analyze the data.

Cell population abundance

The cell populations are plentiful post-sort. Purity was assessed with flow cytometry, confirming a high percentage of target cells. Cell counts exceeded abundance thresholds.

Gating strategy

FSC/SSC were used to discern single cells from doublets/multiple cells. Samples without flourescent staining were used to establish boundaries between negative and positive cells.

- [ ] Tick this box to confirm that a figure exemplifying the gating strategy is provided in the Supplementary Information.
